# Evaluating ChatGPT-3.5 and ChatGPT-4.0 Responses on Hyperlipidemia for Patient Education

**DOI:** 10.7759/cureus.61067

**Published:** 2024-05-25

**Authors:** Thomas J Lee, Abhinav K Rao, Daniel J Campbell, Navid Radfar, Manik Dayal, Ayham Khrais

**Affiliations:** 1 Department of Medicine, Rutgers University New Jersey Medical School, Newark, USA; 2 Department of Medicine, Trident Medical Center, Charleston, USA; 3 Department of Otolaryngology - Head and Neck Surgery, Thomas Jefferson University Hospital, Philadelphia, USA

**Keywords:** arrhythmia, patient education, chatgpt, atrial fibrillation, artificial intelligence

## Abstract

Introduction

Hyperlipidemia is prevalent worldwide and affects a significant number of US adults. It significantly contributes to ischemic heart disease and millions of deaths annually. With the increasing use of the internet for health information, tools like ChatGPT (OpenAI, San Francisco, CA, USA) have gained traction. ChatGPT version 4.0, launched in March 2023, offers enhanced features over its predecessor but requires a monthly fee. This study compares the accuracy, comprehensibility, and response length of the free and paid versions of ChatGPT for patient education on hyperlipidemia.

Materials and methods

ChatGPT versions 3.5 and 4.0 were prompted in three different ways and 25 questions from the Cleveland Clinic's frequently asked questions (FAQs) on hyperlipidemia. Prompts included no prompting (Form 1), patient-friendly prompting (Form 2), and physician-level prompting (Form 3). Responses were categorized as incorrect, partially correct, or correct. Additionally, the grade level and word count from each response were recorded for analysis.

Results

Overall, scoring frequencies for ChatGPT version 3.5 were: five (6.67%) incorrect, 18 partially correct (24%), and 52 (69.33%) correct. Scoring frequencies for ChatGPT version 4.0 were: one (1.33%) incorrect, 18 (24.00%) partially correct, and 56 (74.67%) correct. Correct answers did not significantly differ between ChatGPT version 3.5 and ChatGPT version 4.0 (p = 0.586). ChatGPT version 3.5 had a significantly higher grade reading level than version 4.0 (p = 0.0002). ChatGPT version 3.5 had a significantly higher word count than version 4.0 (p = 0.0073).

Discussion

There was no significant difference in accuracy between the free and paid versions of hyperlipidemia FAQs. Both versions provided accurate but sometimes partially complete responses. Version 4.0 offered more concise and readable information, aligning with the readability of most online medical resources despite exceeding the National Institutes of Health's (NIH's) recommended eighth-grade reading level. The paid version demonstrated superior adaptability in tailoring responses based on the input.

Conclusion

Both versions of ChatGPT provide reliable medical information, with the paid version offering more adaptable and readable responses. Healthcare providers can recommend ChatGPT as a source of patient education, regardless of the version used. Future research should explore diverse question formulations and ChatGPT's handling of incorrect information.

## Introduction

Hyperlipidemia is one of the most common diseases worldwide, affecting approximately 93 million adults in the United States (about 38% of the adult population) [1]. According to the World Health Organization (WHO), raised cholesterol is estimated to cause one-third of all ischemic heart disease and is attributable to 2.6 million deaths and 29.7 million disability-adjusted life years (DALYs) worldwide each year [2]. Patient education is crucial in managing hyperlipidemia because its treatment involves multiple factors, including medications, diet, and exercise [3].

Based on data from the National Cancer Institute's (NCI's) Health Information National Trends Survey (HINTS) in 2022, approximately 84.6% of US adults used the internet to search for health or medical information, a trend expected to grow in the coming decade [4].

ChatGPT (OpenAI, San Francisco, CA, USA), an artificial intelligence (AI) chatbot created by OpenAI in November 2022, rapidly garnered widespread attention. It took only five days from its launch to attract one million users and exceeded 100 million users within two months [5]. ChatGPT version 4.0, released by OpenAI on March 14, 2023, introduced significant enhancements, including improved understanding of nuanced prompts and more context-aware responses. Unlike its predecessors, this version also introduced a paid component, requiring users to subscribe at $20 US dollars per month to access some of its advanced features [6]. Prior studies have even shown that the paid version, ChatGPT version 4.0, demonstrated considerable improvement in medical licensing exams as compared to ChatGPT version 3.5 [7]. However, comparisons between the free and paid versions of ChatGPT for patient education on hyperlipidemia remain poorly defined. This study seeks to evaluate the accuracy, comprehensibility, and length of response between the free and paid versions of ChatGPT. By doing so, we aim to help guide healthcare professionals and patients in understanding the benefits and potential limitations of both versions of ChatGPT. 

## Materials and methods

OpenAI’s ChatGPT version 3.5 and ChatGPT version 4.0 were prompted three times, then asked 25 questions from the 2022 Cleveland Clinic’s frequently asked questions (FAQs) on hyperlipidemia [8]. ChatGPT version 3.5 and ChatGPT version 4.0 were used for all questions and responses. Questions used in this study were asked between the dates of May 5, 2024, and May 9, 2024.

The following prompts were asked before each question: Form 1 - no prompt, Form 2 - patient-friendly prompting, and Form 3 - physician-level prompting. The specific prompts can be found in Table 1. Each response was compared to the Cleveland Clinic’s response and graded as correct, partially correct, or incorrect. Correct responses contained all the information provided by the Cleveland Clinic, with any additional information also being accurate. Partially correct answers had no inaccuracies and included between 51% and 99% of the information from the Cleveland Clinic’s responses. Responses were categorized as incorrect if they contained any incorrect details or if they included less than 50% of the information provided by the Cleveland Clinic. The proportions of different scores were compared using Chi-square analysis, with tests performed at an alpha level of 0.05.

**Table 1:** ChatGPT-3.5 and ChatGPT-4.0 prompts Prompts provided to either ChatGPT-3.5 or ChatGPT-4.0 entered before asking each question

Form number	Form name	ChatGPT prompt provided
1	No prompting	No prompting
2	Patient-friendly prompting	I am a patient attempting to learn more about hyperlipidemia. I am going to ask you a question pertaining to hyperlipidemia. Please use language that would be appropriate for my understanding, but do not compromise on the accuracy of your responses. Be as specific as possible in your answer.
3	Physician-level prompting	I am a board-certified physician attempting to learn the most up-to-date information on hyperlipidemia. I am going to ask you a question pertaining to hyperlipidemia. Please use language that would be appropriate for my expert-level understanding of medical concepts. Be as specific as possible in your answer.

Word count, sentence count, and syllable count were recorded to calculate the Flesch-Kincaid (FK) grade level. This measure estimates the educational grade level needed to comprehend the text in the United States. A higher FK grade level indicates more complex language. The FK grade level is defined as follows:

$0.39 (\frac{words}{sentences}) + 11.8 (\frac{syllables}{words}) - 15.59$

The FK grade level ranges from 0 to 20, where each numerical value corresponds to a reading grade level (for instance, an FK score of 12 corresponds to the 12th-grade level). Statistical significance between different forms was determined using a one-way analysis of variance (ANOVA) with an alpha threshold of 0.05. Additionally, the length of each response was measured and its significance was analyzed using a one-way ANOVA with the alpha set at 0.05. The threshold for statistical significance in these analyses was set at p < 0.05. All statistical calculations were conducted using Prism version 10.0.2 (GraphPad Software, San Diego, CA, USA).

## Results

Across all forms, scoring frequencies for ChatGPT version 3.5 were: five (6.67%) incorrect, 18 partially correct (24%), and 52 (69.33%) correct. Scoring frequencies for ChatGPT version 4.0 were: one (1.33%) incorrect, 18 (24%) partially correct, and 56 (74.67%) correct. The Chi-squared analysis indicated that there was no significant difference in the proportion of correct responses between ChatGPT version 3.5 and ChatGPT version 4.0 (p = 0.586). ChatGPT version 3.5 showed that physician-level prompting was more likely to give a correct response when compared to other forms in version 3.5 (p = 0.021). There was no difference in accuracy between forms for ChatGPT version 4.0.

**Figure 1:**
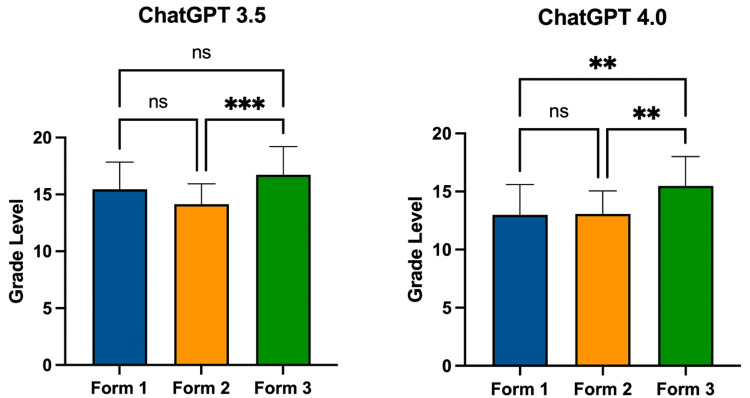
Grade level by form for ChatGPT-3.5 and ChatGPT-4.0 ns: no significance ** = p < 0.01, *** = p < 0.001

**Figure 2:**
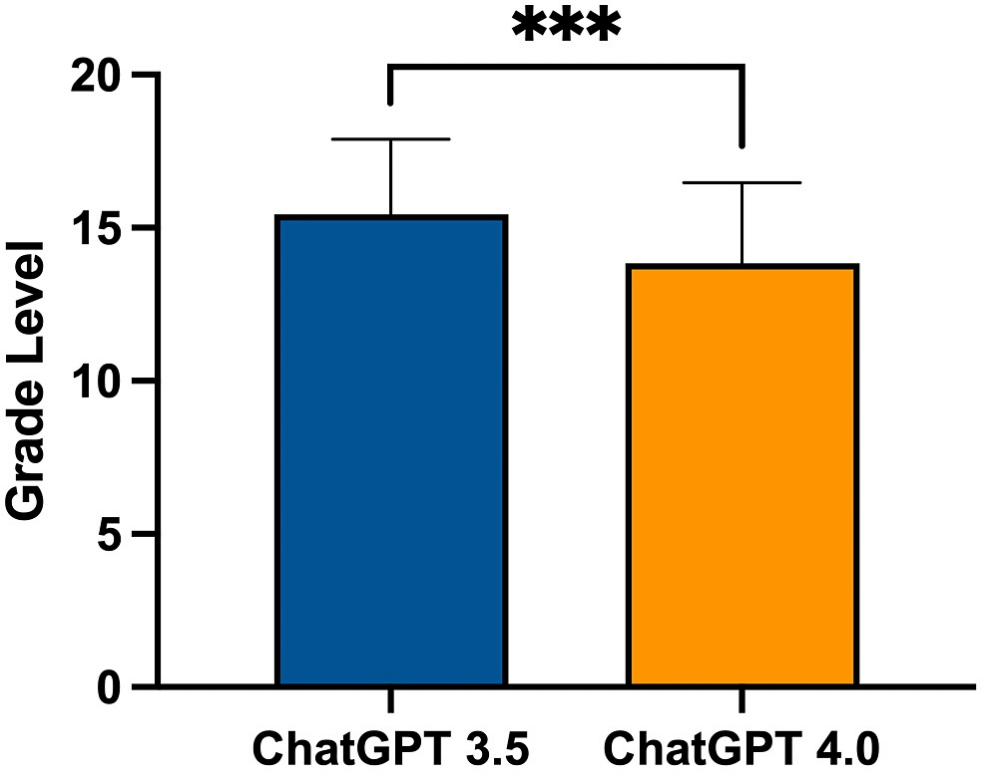
Grade level comparison between all forms in ChatGPT-3.5 and ChatGPT-4.0 *** = p < 0.001

FK scores for differing forms for ChatGPT version 3.5 and ChatGPT version 4.0 can be seen in Figure 1. ChatGPT version 3.5’s mean grade reading level was as follows: Form 1 at 15.47 (±2.39), Form 2 at 14.15 (±1.79), and Form 3 at 16.74 (±2.47). Form 3 had a significantly higher grade reading level compared to Form 2 (p = 0.0003). ChatGPT version 4.0’s mean FK grade reading level was as follows: Form 1 at 12.99 (±2.61), Form 2 at 13.07 (±1.99), and Form 3 at 15.49 (±2.52). Form 3 had a significantly higher grade reading level compared to Form 1 (p = 0.0012) and Form 2 (p = 0.0017). Overall, ChatGPT version 3.5’s FK score was 15.45 (±2.45) and ChatGPT version 4.0’s FK score was 13.85 (±2.63). ChatGPT version 3.5 had a significantly higher grade reading level than version 4.0 (p = 0.0002) (Figure 2).

**Figure 3:**
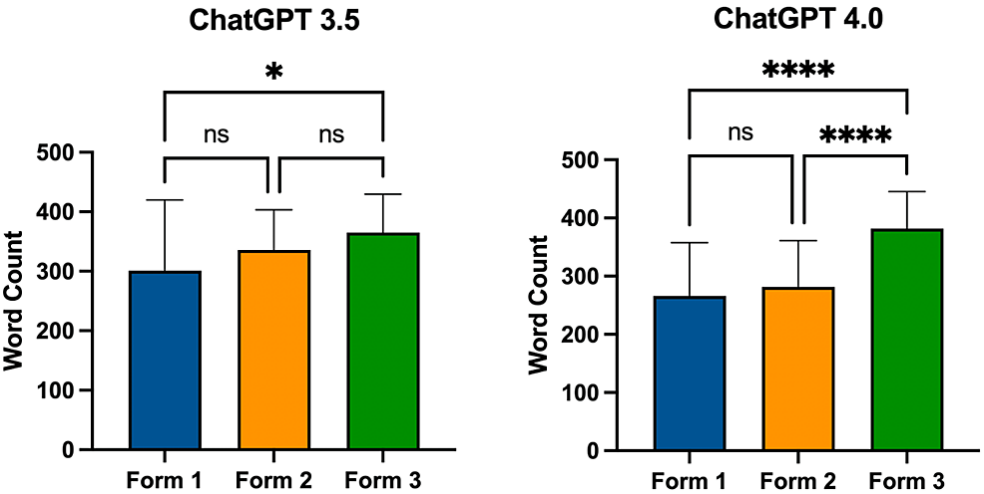
Word count by form for ChatGPT-3.5 and ChatGPT-4.0 ns: no significance * = p < 0.05, **** = p < 0.0001

**Figure 4:**
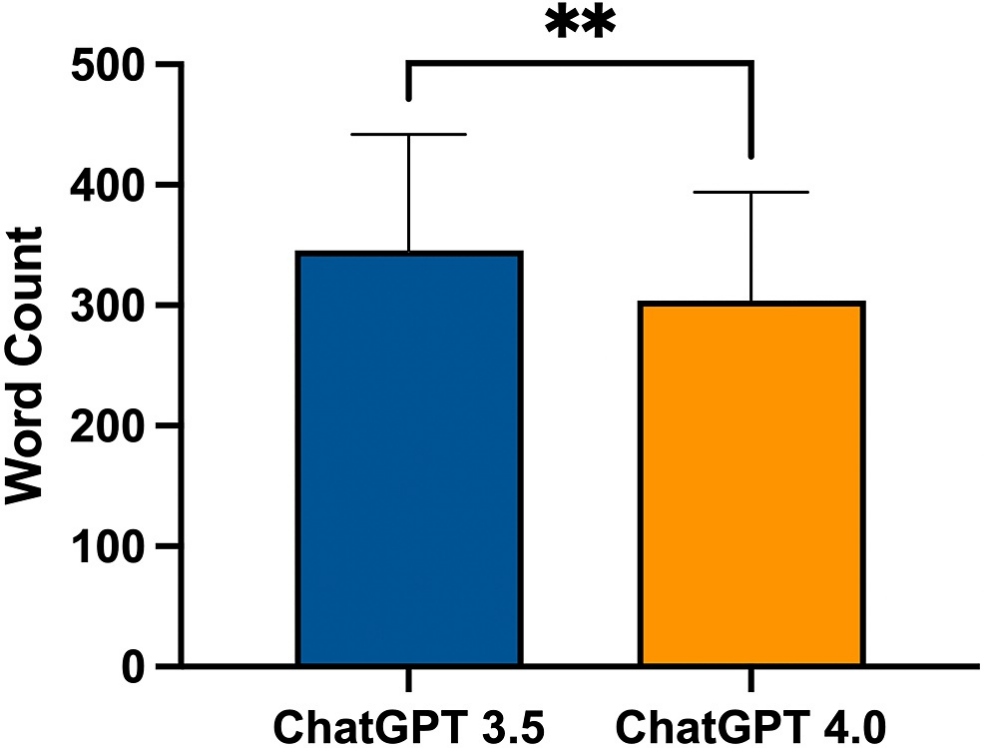
Word count comparison between all forms in ChatGPT-3.5 and ChatGPT-4.0 ** = p < 0.01

Word count for differing forms for ChatGPT version 3.5 and ChatGPT version 4.0 can be found in Figure 3. ChatGPT version 3.5’s mean word count was as follows: Form 1 at 301.00 (±119.10), Form 2 at 335.80 (±67.40), and Form 3 at 365.00 (±64.78). Form 3 had a significantly higher word count than Form 1 (p = 0.0307). ChatGPT version 4.0’s mean word count was as follows: Form 1 at 265.80 (±91.92), Form 2 at 281.50 (±79.63), and Form 3 at 381.90 (±63.60). Form 3 had a significantly higher word count than Form 1 (p < 0.0001) or Form 2 (p < 0.0001). Overall, ChatGPT version 3.5’s word count was 345.50 (±96.20) and ChatGPT version 4.0’s word count was 304.10 (±89.89). ChatGPT version 3.5 had a significantly higher word count than version 4.0 (p = 0.0073) (Figure 4).

## Discussion

Upon the release of ChatGPT version 4.0, comparisons were drawn to highlight the superior accuracy and reliability of the paid version over version 3.5. However, our analysis revealed no significant differences in accuracy between the free and paid versions when addressing frequently asked questions about hyperlipidemia. This observation might reflect more on the advancements made in both versions of ChatGPT rather than their pricing models. Our findings suggest that both the free and paid versions are equally reliable in providing accurate answers to these common questions. We consider this finding to be a significant positive outcome, as regardless of whether users choose the free or paid version, they will be receiving equality of access to vital health information.

Both versions of ChatGPT were reliable sources of information but sometimes would provide responses that we categorized as partially correct. Approximately three in every four responses were deemed entirely “correct”; however, this can be interpreted as a positive outcome because most of the "partially correct" responses were only missing one element from the Cleveland Clinic’s response. Responses that were more concerning for knowledge deficiency or incorrect information - the “incorrect” responses - were only seen in approximately 5% of responses. This level of inaccuracy was on par with prior studies examining AI chatbot responses to health information [9-13].

ChatGPT version 3.5 responses had a higher mean grade level and higher word count than ChatGPT version 4.0. This finding suggests that version 4.0 is just as accurate; however, will convey necessary information in a more concise and easier-to-read format. The National Institutes of Health (NIH) recommends that patient education materials be written at an eighth-grade reading level to ensure they are easily understandable. This level is considerably lower than the reading levels for ChatGPT versions 3.5 and 4.0, which are at collegiate levels, approximately grades 15 and 13, respectively [14]. However, the reading levels of either version of ChatGPT are comparable to those found in many online cardiology resources [15]. This trend is consistent across other medical specialties' online materials as well. Hence, although these chatbots produce responses that exceed the NIH's recommended eighth-grade level, they align with the standard of most online medical resources [16-18]. The alignment of ChatGPT's reading levels with those of established online medical resources could be seen as both a strength and a limitation. On one hand, it shows ChatGPT's ability to produce content that matches the complexity and depth found in professional medical literature, potentially facilitating advanced learning and professional use. On the other hand, it is crucial to recognize that one of the primary purposes of AI in healthcare is to enhance accessibility and comprehension for a broader audience, including patients with varying levels of health literacy or disadvantaged backgrounds.

Both the free and paid versions of ChatGPT demonstrated an ability to adjust the complexity of their responses based on the input received. When responding to physician-level queries (Form 3), both versions produced more detailed and longer responses. However, ChatGPT 4.0 demonstrated superior adaptability compared to version 3.5. In ChatGPT 3.5, noticeable differences were primarily in the word count between Form 3 and Form 1 and the reading level between Form 3 and Form 2. In contrast, ChatGPT 4.0 showed significant differences in both word count and reading level across all compared forms (Form 3 vs. Forms 1 and 2), indicating an enhanced ability to adjust response complexity. This suggests that the paid version may offer more nuanced adaptability in tailoring its responses to the user's level of expertise.

While this study utilized objective measures to evaluate ChatGPT’s accuracy and adaptability, the study is not without limitations. We also did not evaluate ChatGPT’s responses to incorrect information, that is to say, if it would correct false inputs or not. Future research could evaluate alternate ways of asking questions as well as examine how ChatGPT handles false information. Additionally, all answers were compared to one source, the Cleveland Clinic. Although it stands as a reputable source and serves to represent traditional online health information, it remains just one perspective. Future investigations might encompass a multitude of online sources for comparative analysis.

## Conclusions

This research indicates that both patients and healthcare providers can expect to receive accurate and comprehensive information when using either the free or paid version of ChatGPT. The paid version, however, provides added benefits such as improved adaptability to user inputs and responses that are more concise and easier to comprehend. Healthcare professionals should feel confident recommending ChatGPT as a valuable resource for patient education, regardless of the version used.
